# Exploring How the Tobacco Industry Presents and Promotes Itself in Social Media

**DOI:** 10.2196/jmir.3665

**Published:** 2015-01-21

**Authors:** Yunji Liang, Xiaolong Zheng, Daniel Dajun Zeng, Xingshe Zhou, Scott James Leischow, Wingyan Chung

**Affiliations:** ^1^School of Computer ScienceNorthwestern Polytechnical UniversityXi'anChina; ^2^Department of Management Information SystemsEller College of ManagementThe University of ArizonaTucson, AZUnited States; ^3^State Key Laboratory of Management and Control for Complex SystemsInstitute of AutomationChinese Academy of SciencesBeijingChina; ^4^College of MedicineMayo ClinicScottsdale, AZUnited States; ^5^Department of Decision and Information SciencesSchool of Business AdministrationStetson UniversityDeLand, FLUnited States

**Keywords:** cigarette brands, promotional strategy, social media, tobacco control, tobacco promotion

## Abstract

**Background:**

The commercial potential of social media is utilized by tobacco manufacturers and vendors for tobacco promotion online. However, the prevalence and promotional strategies of pro-tobacco content in social media are still not widely understood.

**Objective:**

The goal of this study was to reveal what is presented by the tobacco industry, and how it promotes itself, on social media sites.

**Methods:**

The top 70 popular cigarette brands are divided into two groups according to their retail prices: group H (brands with high retail prices) and group L (brands with low retail prices). Three comprehensive searches were conducted on Facebook, Wikipedia, and YouTube respectively using the top 70 popular cigarette brands as keywords. We identified tobacco-related content including history and culture, product features, health warnings, home page of cigarette brands, and Web-based tobacco shops. Furthermore, we examined the promotional strategies utilized in social media.

**Results:**

According to the data collected from March 3, 2014 to March 10, 2014, 43 of the 70 representative cigarette brands had created 238 Facebook fan pages, 46 cigarette brands were identified in Wikipedia, and there were over 120,000 pro-tobacco videos on YouTube, associated with 61 cigarette brands. The main content presented on the three social media websites differs significantly. Wikipedia focuses on history and culture (67%, 32/48; *P*<.001). Facebook mainly covers history and culture (37%, 16/43; *P*<.001) and major products (35%, 15/43), while YouTube focuses on the features of major tobacco products (79%, 48/61; *P*=.04) and information about Web-based shops (49%, 30/61; *P*=.004). Concerning the content presented by groups H and L, there is no significant difference between the two groups. With regard to the promotional strategies used, sales promotions exist extensively in social media. Sales promotion is more prevalent on YouTube than on the other two sites (64%, 39/61 vs 35%, 15/43; *P*=.004). Generally, the sale promotions of higher-cost brands in social media are more prevalent than those of lower-cost brands (55%, 16/29 vs 7%, 1/14; *P*<.001 for Facebook; 78%, 28/36 vs 44%, 11/25; *P*=.005 for YouTube).

**Conclusions:**

The prevalence of cigarette brands in social media allows more pro-tobacco information to be accessed by online users. This dilemma indicates that corresponding regulations should be established to prevent tobacco promotion in social media.

##  Introduction

To fight against diseases associated with the use of tobacco products—one of the biggest public health threats the world has ever faced—the World Health Organization’s Framework Convention on Tobacco Control introduced a series of measures to implement effective strategies for tobacco control. For example, the packaging and labeling of tobacco products need to carry health warnings describing the harmful effects of tobacco use [1] and terms (such as “low tar”, “light”, or “mild”) that are misleading, deceptive, or likely to create an erroneous impression about tobacco products are prohibited [1].

However, the partial bans prohibiting the advertising and promotion of tobacco products in traditional marketing made the tobacco industry divert to social media with indirect marketing tactics [2]. With more and more people embracing social media sites, such as Facebook and YouTube, the commercial potential of social media with tens of millions of potential consumers connected is emerging. The popularity of social media such as Facebook and YouTube presents the opportunity to raise the visibility of tobacco products and promote tobacco use. A recent study found that British American Tobacco employees were taking advantage of social networking sites to promote the company’s products [3]. On YouTube, many users are exposed to pro-tobacco videos ranging from product reviews to smoking fetish imagery to tobacco-related scenes [4-7]. Obviously, tobacco companies stand to benefit greatly from the marketing potential of social media, without putting themselves at significant risk of being implicated in violating any laws [4].

Many social media sites have policies that outlaw the promotion of tobacco products on their advertising networks. Facebook’s advertising policies claim that ads may not promote or facilitate the sale or consumption of illegal or recreational drugs, tobacco products, or drug or tobacco paraphernalia [8]. Advertisements and commercial content (including Page post content) on Facebook fan pages are subject to these advertising guidelines as well [9]; for YouTube, users are allowed to flag “inappropriate” videos [10]. However, it is still hard to regulate indirect tobacco promotion online.

To regulate tobacco promotion activities in social media, it is necessary to uncover the kinds of information being delivered and how they are presented in social media. This paper examines the top 70 popular cigarette brands and investigates the prevalence of promotion of these cigarette brands in social media. The present study has two specific aims. First, it provides a comprehensive understanding of the kinds of information being delivered in social media for tobacco promotion. Second, it investigates the promotional strategies used. Given the unregulated marketplace for the tobacco industry in social media, useful insights might be gained to inform future regulatory policies by investigating how the tobacco industry presents and promotes itself in social media.

##  Methods

### Data Collection

First, a set of cigarette brands was identified. To maximize the coverage of cigarette brands in our dataset, we collected cigarette brands from three perspectives: tobacco manufacturers, tobacco vendors, and cigarette smokers. According to the US Tobacco Control Act, manufacturers and vendors must be registered and provide a list of tobacco products being manufactured or distributed [11]. Therefore, we choose several official cigarette brand lists [12] and Web-based tobacco shops [13] to cover cigarette brands from the perspective of manufacturers and vendors. On the other hand, for smokers, favorite cigarette brands can be delivered in social media. We integrated the cigarette brands from user-generated data such as tobacco-related wiki webpages [14] and tobacco review websites [15]. This method not only provided many cigarette brands ranging from the multinational tobacco industry such as British American Tobacco to the domestic/local tobacco industry, but also constructed a representative dataset of cigarette brands including the top 10 best-selling cigarette brands in the world [16] and the best-selling cigarette brand in each country [17]. In total, we obtained 186 cigarette brands and counted the number of times that each brand occurs in those data sources respectively. Finally, we chose the representative brands according to the average of occurrence frequency, and got 70 representative brands from the collection of cigarette brands.

In addition, the retail prices of the top 70 cigarette brands were obtained from the price labels found in Web-based tobacco shops. Since there are many variants for each cigarette brand with different retail prices, for this paper we calculated the average retail price of each cigarette. According to the median of average retail prices, these 70 cigarette brands were divided into two groups: brands with high retail prices (group H) and brands with low retail prices (group L). The size of group H is 39, and 31 for group L.

Based on the 70 cigarette brands, three comprehensive searches of cigarette brands were conducted on Facebook, Wikipedia, and YouTube respectively, which are among the top 15 most visited websites in 2014 [18]. For Facebook, we focused on the fan pages named after these cigarette brands. A fan page is a public profile that enables users to share their business and products with Facebook users, where page likes, comments, and sharing are the most common interaction activities [9]. For Wikipedia, we conducted searches using the cigarette brands as keywords and removed websites unrelated to cigarette brands. Due to a huge number of video clips on YouTube, we reviewed the first 20 pages of search results for each brand to capture pro-tobacco video clips.

For Facebook and YouTube, we evaluated whether retrieved results were related to tobacco promotion by examining the page profiles personally. Manual checking of tobacco-related topics was also used in the previous work [4,19-21]. Specifically, the “About” section of profiles is used to determine whether the given fan pages are related to tobacco. The “About” section provides basic information about the fan page, such as the goals of the page, location, operating hours, email address, mobile phone number, and product information. In addition, the website links such as the home pages of the given brands may be provided as well. According to the retrieved results, the majority of them were written in English. However, some non-English content written in Italian, German, French, and even Arabic was obtained as well. For the non-English content, we determined whether or not it was pro-tobacco based on the pictures or videos clips embedded in the posts. For a given fan page on Facebook, if the majority of multimedia content including pictures and video clips was pro-tobacco, the fan page was regarded as a pro-tobacco fan page. For YouTube, if content such as images of young men and women smoking, smoking sexual fetish scenarios, smoking animals or cartoon characters, logos of cigarette brands, and cigarette reviews were presented, the video clip was grouped as pro-tobacco. Content that didn’t show pro-tobacco pictures or videos was excluded directly. For non-English content, sites that didn’t show pro-tobacco pictures or videos directly were excluded. Cigarette brands were coded if we identified at least one cigarette product through a distinct name or logo.

### Measures

The data collection was conducted from March 3, 2014 to March 10, 2014. For the three social media sites, we manually reviewed and classified the user-generated data into five types: history and culture, major products, health warnings, company websites, and Web-based tobacco shops. The content of each type are presented in Table 1.

For Facebook, we reviewed the user-generated data (textual posts, photos, and video clips) and the “About” section of fan pages. In the “About” section of fan pages, the account owner can add different types of basic information about the page, which enables potential followers to quickly learn about the page. For YouTube, we reviewed the “About” section related to video clips as well. To classify the content of video clips, we reviewed video clips manually. The early commercial ads and non-pro-tobacco videos were excluded. For the URLs embedded in video clips, we accessed those websites and classified them as company websites or Web-based tobacco shops.

For tobacco promotional strategies, many researchers have examined how tobacco companies promote themselves. We summarized the most widely utilized strategies from existing literature [3,4,20-23] and analyzed what kinds of methods play important roles in tobacco promotion in social media. Specifically, we investigated the following five promotional strategies: brand promotion, sales promotion, fetish imagery, sponsorship, and misleading information. The descriptions of these five promotional strategies are presented in Table 2. To differentiate brand promotion and sales promotion, we checked the content presented in the profile. Brand promotion covered the following content: product launch time, origin of brand name, ownership, market share, slogan and ads, brand stories, achievements, and even the home pages from the tobacco manufacturing industry presented in social media. For sales promotion, tobacco is promoted with price discounts, coupons, free shipping, no tax, embedded URLs of tobacco shops, etc.

All classifications of content and promotional strategies were conducted according to Tables 1 and 2 by three coders cooperatively. The three coders voted for the categories of the retrieved data. When the three coders disagreed with each other, they discussed the categories and tried to reach consensus. If it was still difficult to achieve an agreement after discussion, the retrieved data was excluded from our dataset.

**Table 1:** Classification of user-generated data in social media.

Content	Description
History and culture	Product launch time; origin of brand name; ownership; market share; slogan and ads; brand stories; manufacturer and distribution location; achievements
Major products	Varieties of products; flavor; packaging; length; tar content, nicotine content, and carbon monoxide content; price
Health warning	Warnings about the side effects of smoking
Company websites	Home pages of tobacco companies
Web-based tobacco shops	URLs of Web-based tobacco shops

**Table 2:** Description of promotional strategies in social media.

Promotional strategies	Description
Brand promotion	Company websites are embedded in the textual data or video clips; content about the history and culture of cigarette brands.
Sales promotion	Promote tobacco sales with price discounts, tobacco coupons, free shipping, no tax, and embedded URLs for tobacco shops, etc.
Fetish imagery	Images of young men and women smoking, smoking sexual fetish scenarios, smoking animals or cartoon characters, etc.
Sponsorship	Provide funds for sports matches, festivals, racing, etc. Eg, Formula One, tennis matches, music bands, and festivals are common places to see logos or brand names from the tobacco industry. In social media, lots of pictures and videos related to social events funded by cigarette brands are presented.
Misleading information	Slogans to smooth or blur the side effects of smoking. Eg, low tar content is emphasized to demonstrate the products are healthier.

## Results

### Classification of Content

As shown in Table 3, in total, 43 cigarette brands had created 238 fan pages on Facebook; 48 cigarette brands created articles on Wikipedia; while for YouTube, more than 120,000 video clips were associated with 61 of the given 70 cigarette brands (87%). The prevalence of cigarette brands in social media demonstrates that the tobacco industry is aware of the commercial potential of social media and embarks on tobacco promotion in social media.

The main content presented on the three social media websites is different. Wikipedia has a different purpose than the other two sites. Wikipedia mainly focuses on brand promotion with history and culture (67%, 32/48; *P*<.001) and major product features (56%, 27/48) instead of sales promotion (4%, 2/48); Facebook mainly focuses on history and culture (37%, 16/43; *P*<.001) and major products (35%, 15/43); while YouTube focuses on major products (79%, 48/61; *P*=.04) and Web-based shops (49%, 30/61; *P*=.004). Obviously, the content concerning major products plays an important role in social media to promote tobacco products. Many details about tobacco products including tobacco flavor, package, tar content, and varieties are presented, which may help potential buyers to quickly learn about product features.

Regulations about the health warnings on cigarette packaging are enforced by many countries. The health warning labels on cigarette packaging illustrate the health dangers of tobacco products. However, as shown in Table 3, the side effects of smoking are rarely presented in social media. By contrast, the home pages of tobacco corporations or Web-based tobacco shops are presented frequently. Tobacco sales promotions exist extensively on YouTube with embedded URLs of Web-based tobacco shops (49%, 30/61); 26% (11/43) for Facebook. The embedded URLs make it more convenient for potential buyers to access those websites and result in brand loyalty and tobacco consumption. Concerning the content presented in groups with high and low retail prices, there is no significant difference between the two groups (see Table 4).

**Table 3:** Classification of pro-tobacco content on Facebook, Wikipedia, and YouTube.

	Facebook	Wikipedia	YouTube	*P* value		
				Facebook vs Wikipedia	Wikipedia vs YouTube	Facebook vs YouTube
Number of brands	43	48	61			
History and culture, n (%)	16 (37)	32 (67)	5 (8)	<.001	<.001	.001
Major products, n (%)	15 (35)	27 (56)	48 (79)	.04	.03	<.001
Health warnings, n (%)	1 (2)	3 (6)	2 (3)	.04	.95	.47
Web-based tobacco shops, n (%)	11 (26)	2 (4)	30 (49)	.004	<.001	.004
Company websites, n (%)	11 (26)	11 (23)	4 (7)	.77	.02	.009

**Table 4:** Differences in content presented in two tobacco groups^a^.

	Facebook (n=43)			Wikipedia (n=48)			YouTube (n=61)		
	H	L	*P* value	H	L	*P* value	H	L	*P* value
Number of brands	29	14		31	17		36	25	
History and culture, n (%)	13 (46)	3 (21)	.10	22 (71)	9 (53)	.40	3 (8)	3 (12)	.97
Major products, n (%)	14 (49)	2 (14)	.01	20 (65)	8 (47)	.24	31 (86)	18 (72)	.30
Health warnings, n (%)	1 (3)	1 (7)	.31	2 (6)	1 (6)	.94	2 (6)	1 (4)	.78
Online tobacco shops, n (%)	10 (34)	2 (14)	.12	2 (6)	0 (0)	.14	19 (53)	11 (44)	.32
Company websites, n (%)	10 (34)	2 (14)	.12	8 (26)	3 (18)	.50	5 (14)	0 (0)	.02

^a^Group H (brands with high retail prices) and Group L (brands with low retail prices).

### Promotional Strategies

Table 5 shows the tobacco promotional strategies utilized by these 70 cigarette brands in social media. Company website, fetish imagery, and sponsorship are more widely utilized for brand promotion; while sales promotion plays an important role in tobacco sales campaigns. In particular, URLs of Web-based tobacco shops are frequently embedded in the posts and profiles for sales promotions. For example, Winston, Black Devil, and Camel cigarettes provided the links of Web-based cigarette shops. Even though it is difficult to determine how many users accessed those given links, it is obvious that those links make it convenient for smokers to purchase cigarettes.

According to Table 5, sales promotion is more prevalent on YouTube with a share of 64% (39/61). The sales strategies include embedded URLs of online tobacco shops (49%, 30/61) and other sales methods including price discounts, tobacco coupons, and free shipping. Similarly, sales promotion is often adopted by Facebook as well (35%, 15/43). Furthermore, the tobacco industry misleads potential users with statements that their products are less harmful to one’s health. Many brands use terms (such as “low tar”, “light”, “mild”) to blur the numerous side effects and to create an erroneous impression about tobacco products by emphasizing the low tar content, nicotine content, and carbon monoxide content. For example, American Spirit marketed its products as being “100% Additive-Free Tobacco” and as being less radioactive cigarettes with organic tobacco. Many manufacturers provide product variants with reduced tar. In addition, the tobacco industry tends to promote cigarette brands with sponsorships of social events such as Formula One racing, music festivals, marathons, etc.

For sales promotion, tobacco industries also try to persuade users on Facebook to buy cigarettes, with many different approaches: (1) the administrators of fan pages use postings to lure followers to buy cigarettes online, and (2) some Web-based tobacco shops such as “cheap cigarette 4 you”, “foreign cigarettes”, and “buy cigarettes online Canada and USA” were linked with cigarette brands. Even the links directing to Web-based cigarette shops are posted on fan pages, which makes it easier for potential buyers to purchase cigarettes online by clicking on those links directly.

Compared with less expensive cigarette brands, expensive brands are more successful in brand promotion and sales promotion (see Table 6). They are more likely to embed home pages in Facebook pages (34%, 10/29 vs 14%, 2/14). More importantly, the sales promotions of expensive brands in social media are more prevalent than those of less expensive brands (55%, 16/29 vs 7%, 1/14; *P*<.001 for Facebook; 78%, 28/36 vs 44%, 11/25; *P*=.005 for YouTube). This implies that the marketing channels for expensive brands are more flexible and their distribution networks are more powerful.

**Table 5:** Comparison of promotional strategies on Facebook, Wikipedia, and YouTube.

	Facebook	Wikipedia	YouTube	*P* value		
				Facebook vs Wikipedia	Wikipedia vs YouTube	Facebook vs YouTube
Number of brands	43	48	61			
Brand promotion, n (%)	11 (26)	11 (23)	4 (7)	.77	.02	.009
Sales promotion, n (%)	15 (35)	2 (4)	39 (64)	<.001	<.001	.004
Fetish imagery, n (%)	11 (26)	2 (4)	22 (36)	.003	<.001	.25
Sponsorship, n (%)	4 (9)	9 (19)	6 (10)	.19	.12	.93
Misleading, n (%)	6 (14)	7 (15)	4 (7)	.94	.18	.23

**Table 6:** Differences of promotional strategies in two tobacco groups^a^.

	Facebook			Wikipedia			YouTube		
	H	L	*P*	H	L	*P*	H	L	*P*
Number of brands	29	14		31	17		36	25	
Brand promotion (%)	10 (34)	2 (14)	.12	8 (26)	3 (18)	.50	5 (14)	0 (0)	.02
Sales promotion (%)	16 (55)	1 (7)	<.001	2 (6)	0 (0)	.14	28 (78)	11 (44)	.005
Fetish imagery (%)	10 (34)	2 (14)	.12	2 (6)	0 (0)	.14	16 (44)	7 (28)	.09
Sponsorship (%)	3 (10)	1 (7)	.72	7 (23)	3 (18)	.87	5 (14)	2 (8)	.46
Misleading (%)	5 (17)	1 (7)	.31	6 (19)	2 (12)	.47	5 (14)	0 (0)	.02

^a^Group H (brands with high retail prices) and Group L (brands with low retail prices).

##  Discussion

### Principal Findings

The intent of this study was to reveal what the tobacco industry presents and how it promotes itself on social media sites including Facebook, YouTube, and Wikipedia. Our findings show that the main content presented on the three social media websites is different. Wikipedia focuses on history and culture (67%, 32/48; *P*<.001). Facebook mainly focuses on history and culture (37%, 16/43; *P*<.001) and major products (35%, 15/43); while YouTube focuses on the features of major tobacco products (79%, 48/61; *P*=.04) and information about Web-based tobacco shops (49%, 30/61; *P*=.004). With regard to promotional strategies, sales promotions occur extensively in social media. Sales promotion is more prevalent on YouTube (64%, 39/61 vs 35%, 15/43; *P*=.004). Generally, the sale promotions of higher cost brands in social media are more prevalent than those of inexpensive brands (55%, 16/29 vs 7%, 1/14; *P*<.001 for Facebook; 78%, 28/36 vs 44%, 11/25; *P*=.005 for YouTube). This implies that the marketing channels for expensive brands are more flexible and their distribution networks are more powerful.

### Prevalence of Cigarette Brands

**Figure 1:**
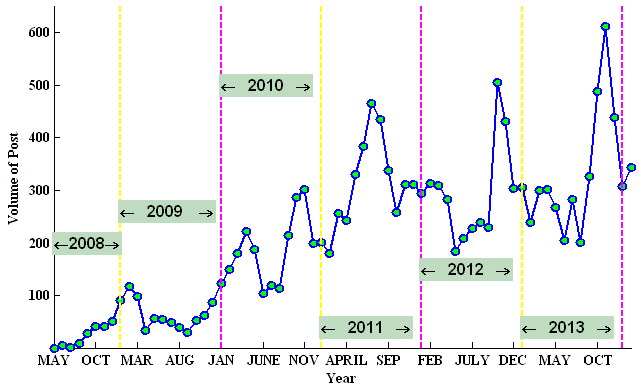
Temporal patterns of post volume on tobacco brand-related fan pages.

The popularity of social media such as Facebook, YouTube, and Wikipedia has provided the opportunity for the tobacco industry to raise the visibility of tobacco products and promote tobacco use. On Facebook, 43 of the 70 cigarette brands have created 238 fan pages with 1,189,976 page likes and 19,022 posts. As shown in Figure 1, the post volume on fan pages is increasing steadily. The accumulating pro-tobacco content on Facebook poses a challenge for tobacco control. Although it is difficult to evaluate the impact of pro-tobacco content for starting or developing smoking habits, the potential is there due to the large number of Facebook users exposed to pro-tobacco content.

**Figure 2:**
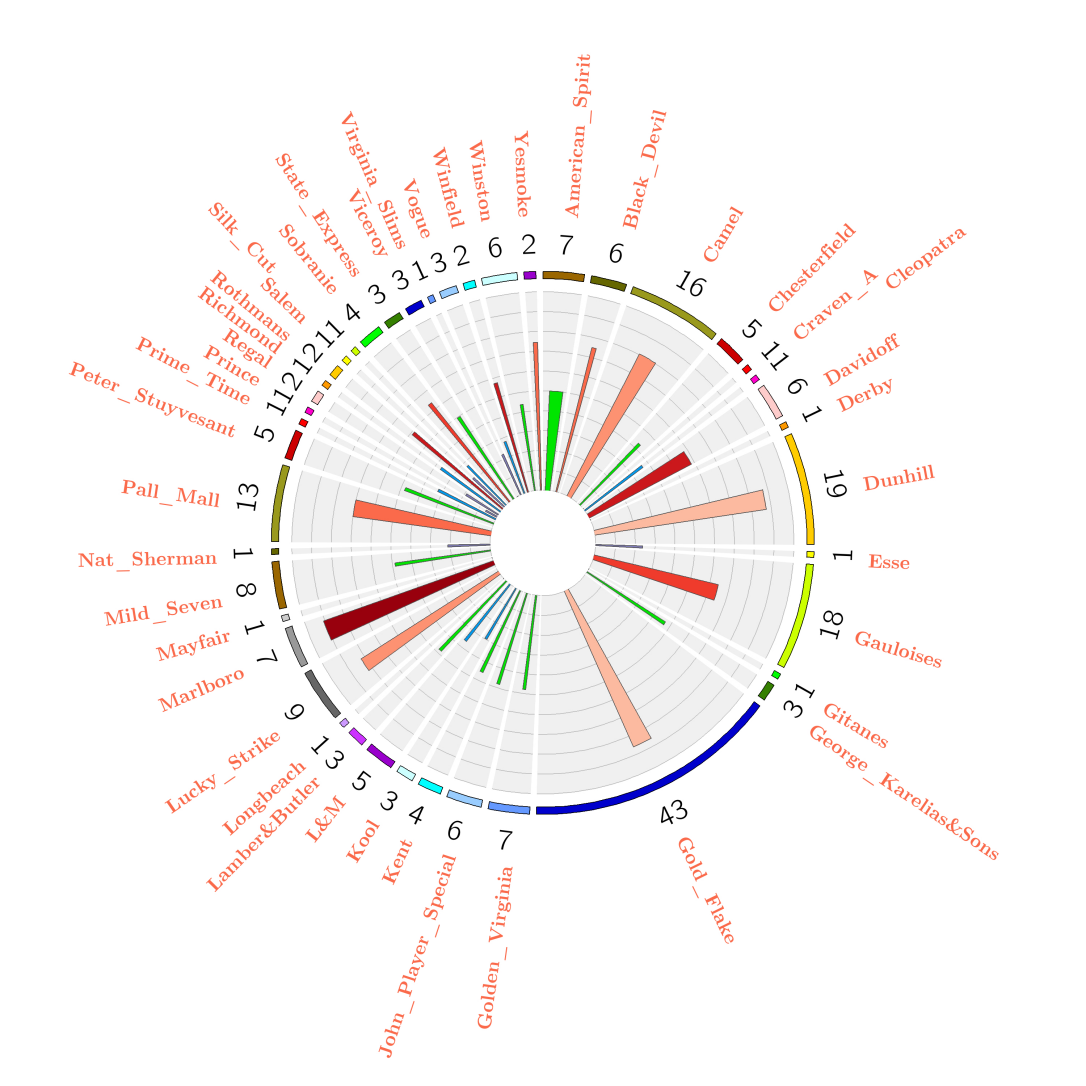
Statistical features of tobacco brands on Facebook.

The statistical features of cigarette brands presented on Facebook are presented in Figure 2. The exterior labels illustrate all 43 cigarette brands on Facebook. The numbered ring indicates how many fan pages are named after the given cigarette brand. For example, 43 fan pages are named after Gold Flake; while Camel, Dunhill, Gauloises, and Pall Mall closely follow with over 10 fan pages. Pertaining to page likes, Lucky Strike overwhelms other brands with, in total, 172,862 page likes on 9 fan pages. Dunhill, Black Devil, and Camel are also popular cigarette brands with 120,696, 63,758, and 44,355 Facebook page likes respectively. In terms of post volume, Gold Flake, Lucky Strike, and Dunhill are the top 3 brands with 2981, 2651, and 2592 posts respectively.

Furthermore, we measured whether or not user interaction is active on the given fan pages by information entropy [24], which was originally introduced from information theory, where entropy is defined as the average amount of information contained in each message and is understood as a measure of uncertainty. In this paper, we use entropy to measure the uncertainty of user interaction in online tobacco-related communities. The users with higher entropy are regarded as active users and may have more powerful influence on the followers. In this paper, the term “interaction entropy” is equal to “entropy”. We use different colors to present the interaction entropy for a given cigarette brand. According to the interior sectors in Figure 2, Marlboro, Dunhill, Gold Flake, and Lucky Strike are the top 4 brands with higher interaction entropy. This demonstrates that Marlboro is the most active cigarette brand on Facebook to interact with Facebook users. Obviously, cigarette brands on Facebook succeeded in attracting Facebook users to interact on fan pages with latent brand promotion and sales promotion.

Furthermore, many fan pages try to enhance user engagement with multiple strategies, for example, attracting users with positive impressions. A page named “animal smoking durrys” posts a huge number of pictures that present smoking animals. This attracts many users to interact with this page via likes, comments, and reposting. Also, images of young men and women smoking are utilized to promote the idea that smoking is cool and fashionable. In addition, online user surveys were conducted on those fan pages. Questions such as “What made you start smoking and at what age?” and “Would you rather die young and smoke durries or have a long life without durries?” were posted and attracted hundreds of followers participating in the online interaction, thus increasing user engagement.

**Figure 3:**
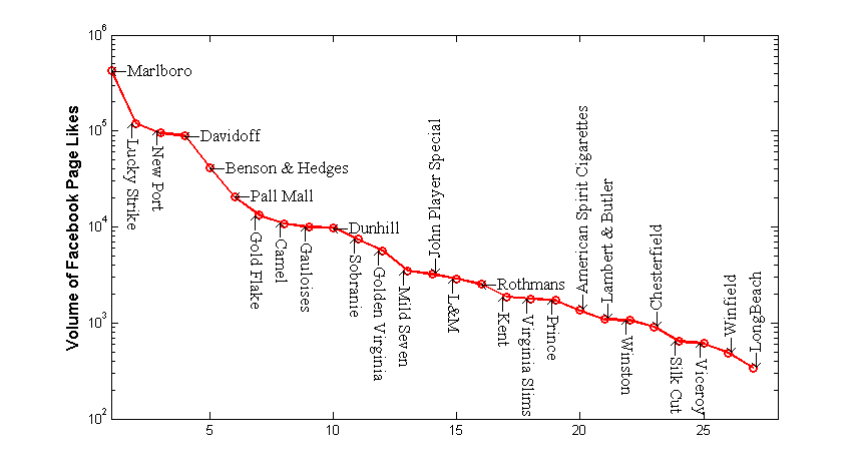
Distribution of user likes for Wikipedia webpages.

Additionally, many cigarette brand-related articles in Wikipedia could be accessed by Facebook users by Facebook likes. These articles cover lots of tobacco-related information such as history, brand culture, major products, etc. We analyzed the volume of page likes on these articles. In total, 876,183 Facebook users liked these 43 cigarette brands sourced from Wikipedia. As shown in Figure 3, Marlboro, followed by Lucky Strike with 119,185 and NewPort with 95,777, is ranked first with 428,646 Facebook fans. Davidoff and Benson & Hedges are ranked in the top 5 popular brands in terms of volume of fans as well. First, the wiki open platform provides an opportunity for the tobacco industry to promote corporation history and culture. Therefore, many articles are created by anonymous volunteers as a reliable source of crowd knowledge [25]. Second, a huge number of Internet users are exposed to cigarette brand-related articles. Even though the impact of articles on potential smokers is difficult to quantify, the aggregation effects are obvious with a large number of users in favor of these cigarette brands.

Through the analysis of user interaction on Facebook and the distribution of page likes for cigarette brand-related articles on Wikipedia, we aim to provide evidence to show the prevalence of cigarette brands in social media. With the popularity of social media such as Facebook, YouTube, and Wikipedia, a huge number of users are exposed to pro-tobacco content and even interact with such information. Obviously, the tobacco industry utilizes social media to raise the visibility of tobacco products and promote tobacco use. As exposure to tobacco promotion is positively associated with a higher smoking prevalence and may encourage smoking initiation [26], regulations about tobacco control in social media should be enforced as soon as possible by decision makers.

### Potential Methods

Through the analysis of Facebook fan pages, we found 61% of brands (43/70) are dedicated to tobacco promotion, especially for international cigarette brands such as Pall Mall and Mild Seven. Furthermore, a surging number of Facebook users have accessed and interacted with these fan pages. Although it is difficult to quantify how those pro-tobacco pages impact users’ attitudes toward tobacco products, we are aware that the presence of cigarette brands in social media is recognized by the tobacco industry or pro-tobacco agencies. Freeman et al [3] investigated two cigarette brands, Dunhill and Lucky Strike, on Facebook. In their literature, there were 44 Facebook fan pages related to Lucky Strike with 28,309 fans in total. For Dunhill, it was 6 pages with 1903 fans. In our study, we found that there were 172,862 fans on 22 pages for Lucky Strike and 120,696 fans on 9 pages for Dunhill respectively. Although some Facebook fan pages have been deleted by their creators or administrators, the volume of fans who like those two cigarette brands has rapidly grown. The explosive growth of page fans demonstrates that social media has played an important role in disseminating cigarette brands.

Our findings also exhibit the loopholes in the mission of tobacco control. Many social media services have policies that outlaw the promotion of tobacco products on their advertising networks. However, those policies do not work well as they require seamless monitoring of content on those websites. The lack of consistent regulation of tobacco promotion, whether directly or indirectly, in social media means that pro-tobacco information is likely to be accessed and shared by anyone, anywhere, no matter what age.

From a legal perspective, we are still at a preliminary stage for tobacco control in social media. Although there is a tobacco advertising ban for social media, it mainly focuses on the advertising that appears as click-through advertisements that display on the sidebar of website pages. With the explosive growth of user-generated pro-tobacco content, we should pay more attention to the content with potential effects for tobacco promotion, as pro-smoking content online, regardless of whether it is commercial or personal in origin, could equally influence users [21].

From a technical standpoint, an online pro-tobacco content surveillance system is needed for the automatic collection and analysis of content relevant to tobacco [27]. With the progress of tobacco wars in social media, seamless online tobacco surveillance is vitally important to properly assess the current situation, the potential risks, and the kind of countermeasures to be taken. In addition, identification of pro-tobacco information in social media is crucial. Technically, the progress of text mining will assist in the discovery of potential pro-tobacco content. Text mining could be utilized to find tobacco-related postings from large-scale user-generated content and sentiment analysis could be employed to uncover the emotions toward tobacco. In addition, to distinguish user postings from industry postings, social media analysis could be introduced to find topological patterns such as clustering coefficients and popularity. Furthermore, social media should be utilized for tobacco control campaigns and tobacco cessation services. The cascade model of information dissemination in social media will help tobacco control campaigns to access hard-to-reach populations [28,29]. Social media can also be adopted for health promotion interventions [30].
